# The Role of Very Low Level Blast Overpressure in Symptomatology

**DOI:** 10.3389/fneur.2019.00891

**Published:** 2019-08-28

**Authors:** Venkata Siva Sai Sujith Sajja, Christina LaValle, Jonathan E. Salib, Anthony C. Misistia, Meron Y. Ghebremedhin, Alejandro N. Ramos, Michael Joseph Egnoto, Joseph B. Long, Gary H. Kamimori

**Affiliations:** Blast-Induced NeuroTrauma Branch, Walter Reed Army Institute of Research, Silver Spring, MD, United States

**Keywords:** sound pressure, blast, overpressure, mTBI, bTBI, symptomology

## Abstract

Blast overpressure exposure has been linked to transient, but measurably deteriorated performance and symptomatologies in law enforcement and military personnel. Overlapping sub-concussive symptomatology associated with the very low level blast overpressures (vLLB) but high sound pressure (<3 psi) associated with these exposures has largely been ignored. Notably, the current vLLB or acoustic literature has focused exclusively on auditory defects, and has not addressed the broader concerns of Soldier health and readiness. This work was prompted by reports of symptomatology such as headache, nausea, slowed reaction time, and balance/hearing complications among personnel undergoing frequent exposures to low overpressure accompanied by high acoustic pressures. To more fully address the consequences associated with low overpressure exposures (<3 psi), a pilot proof-of-concept study was implemented, and data was acquired at two sites on the Fort Benning grenade course range. Findings indicated overpressures ranged from 0.14 to 0.42 psi (0.97–2.89 kPa) at range 1 and 0.22–0.30 psi (1.52–2.07 kPa) on range 2 of the grenade course. Corresponding sound-meter data varied from 153.72 to 163.22 dBP. Headache and long think were the most frequently reported symptoms (3/6 instructors), with lightheadedness, ringing of the ears, restlessness, frustration, and irritability also increasing in 2/6 of the instructors post exposure. Long think (prolonged thinking), ringing of the ears, restlessness, and irritability were the most severe symptoms, with the highest reported post exposure value rating a 3 on the 0–4-point scale. We demonstrate that low-level repeated overpressure exposure can result in transient symptomatology that overlaps with sub-concussive like effects.

## Introduction and Background

The consequences of blast overpressure (OP) exposure typically include transient, concussion-like effects (1) resulting in deteriorated performance (2). These symptoms are sometimes referred to as “Breacher's Brain” (3, 4). The majority of training exposures to blast for military and law enforcement personnel are often characterized as low OP exposure. However, the largely-overlooked very low level blast overpressures (vLLB) (sound pressure) resulting from these exposures may also affect Soldier health and readiness. It is possible that the ephemeral blast effects on Soldiers' performance described in the current body of literature can partially be attributed to acoustic influences. This work was prompted by reports of headache, nausea, slowed reaction time, and balance/hearing complications observed among personnel in routinely low OP but high acoustic exposure training environments. These symptoms have been previously linked to blast exposure (5, 6), but these earlier studies lacked simultaneous sound pressure recordings (i.e., acoustic information), and had exposures relatively higher than the OP recorded with the exposures being reported in the current study. This work seeks to contribute to the evolving understanding of how vLLB affects warfighters in operational training.

The sub-clinical effects of blast exposure (collectively referred to as breachers' brain) include symptomatologies such as sleep disturbances, slow reaction time (long think) and nausea that are thought to be the consequences of brain perturbations. These underlying perturbations are being investigated as part of a growing body of research focused on blast exposure (3, 4, 7–10). In these studies, blast exposures have been predominantly characterized by the peak amplitude(s), or occasionally by the total impulse experienced by a subject over a given set of exposures. The absence of any accompanying sound pressure/acoustic integration is somewhat surprising. The described role of the ear mediating sub-concussive symptomatology such as tinnitus, headache, and hearing issues is well-documented. Researchers have previously noted that acoustic evaluation of overpressure is a reliable indicator as part of OP assessments and can even provide insight into weapon characteristics (11). In addition, the weapons systems can yield high acoustic signatures that can have effects not only on hearing, but also on systemic equilibrium, organ damage, and other negative consequences (12). Though these and other studies have pointed to the non-trivial impact of vLLB associated with the high sound signatures accompanying OP, the health research has been notably underdeveloped in tracking this element of potential health hazards.

Investigations into acoustics note that virtually all common man-portable munitions fielded by NATO members produce acoustic signatures between 145 and 190 dB (13). The high levels of acoustic exposure commonly resulting from these munitions place users at high risk for acoustic damage. Though previous investigations have evaluated the pressure/acoustic intersection of various weapon systems and their consequence to hearing (14, 15), the link to more expansive symptomatology is largely unexplored. The objective of this study is to characterize vLLB or acoustic pressures from commonly deployed munitions (which yield minimally detectable OP exposure as measured with traditional blast sensors) and determine if these vLLB/acoustic pressure exposures produce the same symptoms as are seen with traditional OP exposures (Breachers' Brain) of military personnel.

## Methods

**Figure 1:**
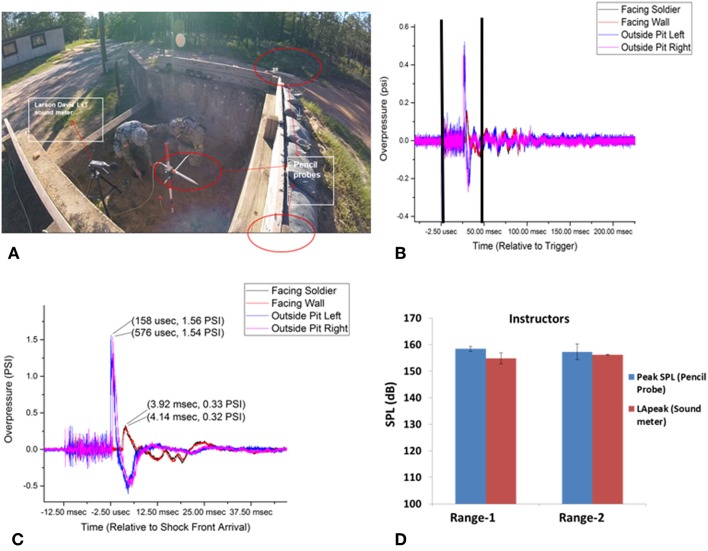
**(A)** Showing the instrumentation location inside a pit of range-1. **(B)** Pencil probes recording of the pressure at different locations of range-1. **(C)** Zoomed in version (area of the black bars in panel **B**) of the oncoming pressure wave recorded at different locations in the grenade pit of range-1. **(D)** Sound pressures recorded in range-1 and range-2 of sound meter and pencil probes, no significant differences were observed when both sensors types are compared.

All subjects that were recruited had consented to participate in the study, and the human use protocol for the interaction with the subjects was approved by Human Subjects Protection Branch of the Walter Reed Army Institute of Research (Silver Spring, MD) and chains of command prior to data collection. The procedures were followed in accordance with the ethical standards of the IRB and the Helsinki Declaration. Target subjects for this preliminary study were grenade range instructors. The characterization of this data collection consists of three parts—blast assessment, acoustic assessment, and personnel assessment. Data were collected over 1 day at two training sites situated at Ft. Benning, GA. Acoustic and blast assessments are the collective characterization of 130 grenade detonations at multiple pits across both locations at Ft. Benning, and symptom reporting consists of the input from the instructors who chose to participate in the study (all instructors operating in the pits elected to participate, *n* = 6). The two ranges were instrumented with pencil probes and sound meters in accordance with MIL-STD-1474E (Figure 1A for an overview of range construction). Participants wore double ear protection (in and over ear) during participation in this study.

### OP Assessment

Two types of blast gauges were deployed. Pencil probes (Figure 1A) were deployed at three locations in this experiment; in the pit, immediately adjacent to the subjects (oriented for incident blast measurement to the detonation area), and at the front of the pit in line with the top of the pit wall (oriented for incident and full reflective pressure measurements, respectively) recorded at 800,000 Hz sampling rate. Additionally, four Blackbox Biometric sensors were placed on the subjects with one on each shoulder, and one on the left/right side of the helmet, all oriented such that the sensors would read incident pressure relative to the direction the subject were facing blast OP.

### Acoustic/Sound Pressure Assessment

Acoustic sensors (Larsron Davis LxT sensors 25,000 Hz Sample Rate, Figure 1A) were mounted to the individuals in the pit on the rear of their right shoulder (near the scapula), which did not impede movement or throwing motion. When the subjects sought cover after the grenade was thrown, the entire device (along with the subject) was completely behind the front wall of the grenade pit. This was done to help ensure exposures are not over-representative of the experienced acoustic effects.

Personnel Assessment Pre and post personnel assessments were conducted over a single day of data collection (prior to blast/training, end of day/training) using previously established symptom metrics (3, 9, 10, 16) that employed a five-point likert-type scale ranging from 0- did not experience the symptom at all, to 4- a severe problem—constantly present, feels like it could affect individual's performance. Subjects were all male and averaged 30.34 + 4.93 years old (min 24, max 36) and 11.5 + 4.92 years of service (min 6, max 15.5). Symptoms were further assessed by asking them if the symptom was experienced constantly or intermittently. The measure is an expanded version of the Neurobehavioral Symptom Inventory (NSI) and Rivermead post-oncussive survey questionnaire. Symptoms reporting focused on headaches, feelings of dizziness, nausea, sleep disturbance, fatigue, mood, cognitive processing via questions assessing concentration, speed of thinking, memory, and hearing complications. Change in symptom was reported for only the increased difference between pre and post-test assessment in the training personnel.

## Results

### OP Assessment

Starting first with the assessment of OP, results were consistent across both ranges investigated in terms of overpressure experienced (psi) and acoustic dB exposure. Pressure readings reported from the pencil probes (representative pressure profiles are shown in Figures 1B,C) ranged from 0.14 to 0.42 psi (0.97–2.89 kPa) at range 1 and 0.22–0.30 psi (1.52–2.07 kPa) on the range 2 (Figure 1D). B3 blast gauge readings were not reported because the pressure exposures did not trigger the sensors.

### Acoustic/Sound Pressure Assessment

When evaluated in terms of peak acoustic dB, range 1 exposures varied from 153.72 to 163.22 dBP and from 157.59 to 160.26 dBP on range 2. The trace of blast events, the pressure wave of the event, and the ranges of the associated acoustic signatures are shown in Figures 1B–D.

### Personnel Assessment

Symptomatology was evaluated with pre/post grenade range exposure of the six range instructors present for the training. Headache and long think (the phenomenon where processing of information is impeded) were the most frequently reported symptoms, increasing in 3 out of 6 the instructors after grenade exposure. Lightheadedness, ringing of the ears, restlessness, frustration, and irritability also increased in 2 out of 6 the instructors' post exposure. Long think, ringing of the ears, restlessness, and irritability were the most severe symptoms, with the highest reported post exposure value rating a three on the 0–4-point scale. Most of these high scores were reported as intermittent, but ringing of the ears was most often reported as a constant problem.

Notably, most instructors had symptoms at baseline that were exacerbated post-exposure. Though new symptom rates varied between 0 and 33% per type of symptom, it is noteworthy that these effects are most often reported at baseline and are then modified post-OP exposure, and all reported symptoms here increased in severity—meaning the instructors were more symptomatic post exposure, and symptoms increased in negative consequence for 1/3 to ½ of instructors across the symptoms reported here for each symptom (Table 1). Looking at variation from pre to post-test, all average reported symptoms increase except restlessness. Restlessness increased in the highest reported intensity, but not in the overall average rate across the instructors.

**Table 1:** Analysis of symptom reporting and exposure.

**Symptom**	**Pre-test avg**	**Post-test avg**	**H/L severity (pre)**	**H/L severity (post)**	**Proportion affected (pre)**	**Proportion affected (post)**
Headache	1.0	1.3	0, 2	0, 2	4/6	5/6
Long think	0.5	0.8	0, 2	0, 3	2/6	3/6
Lightheadedness	0.3	0.7	0, 1	0, 2	2/6	2/6
Ringing of the ears	1.3	1.3	0, 2	0, 3	5/6	4/6^*^
Restless	1.3	1.0	0, 2	0, 3	5/6	3/6
Frustrated	1.0	1.2	0, 2	0, 2	3/6	5/6
Irritable	1.3	1.8	0, 3	0, 3	5/6	5/6

*Summary of pre-post-symptom reporting. N = 6, items on 5 pt lickert scale*.

* *Note—even though one less person reported ringing of the ears on severity, all participants pre and post reported either intermittent or constant ringing of the ears. Headache symptoms were same before and after, plus 1 new subject. Long think had 1 subject stop symptoms, and 2 new subjects report symptoms. Lightheadedness had 1 subject stop reporting, and 1 new subject start reporting. Ringing of the ears had the same subjects minus 1 report, restless was same subjects minus 2. Frustrated was the same subjects plus new subjects. Irritability was the same subjects before and after*.

Taking the findings from blast, acoustic, and personnel assessments in total, results indicate that acoustic pressure, even when combined with limited OP exposure, has associated symptomatologies that manifest like breachers' brain.

## Conclusion/Summary

This work contributes to a growing body of literature ascertaining the extent to which OP exposure above certain thresholds may heighten risks to exposed personnel. Previous investigations had focused on the nature of the blast wave itself, primarily in the form of peak OP and impulse experienced by the subject. These blast-focused efforts largely ignored the potentiality of acoustic pressure as a contributor to OP symptoms. Additionally, several previous efforts did not identify the changes seen here, despite working with a similar population (17, 18). An important distinction between this effort and the aforementioned works (17, 18) is the frequency of exposure. Those efforts used samples of “breachers”—commonly used participants in blast OP research. However, for many training cycles their exposure frequency is quite low— <5 blasts per day. The population sampled here experienced exposures an order of magnitude more frequently. The increased frequency of high acoustic exposure makes the distinction between these groups meaningful. Frustration and headache increased on average score, but did not increase on highest reported level of complaint. The remaining symptoms increased both in severity as well as in overall average.

We found that significant acoustic exposure with corresponding low OP exposure (<1 psi) during military training exercises that may contribute to breachers' brain-like symptoms in instructors, and that measured acoustic signatures are substantial despite current personal protective equipment usage. Anecdotally, adherence to personnel protective equipment (PPE) usage may not be consistent across units and personnel, and is a point of further investigation. This data is at odds with previous findings from a breacher population, however, the total number of exposures (2–4 maximum) are far fewer in the breacher setting on a given day (16). Without the information about the exposure conditions, such as OP or acoustic pressure and stand-offs distances, it is hard to make meaningful comparisons. In addition, the breacher population tends to stand 2–3 times the mean safe distance in the Quantico breaching school to mitigate the effects from breaching exposures (16).

We speculate that the acoustic pressure is the primary contributor to the symptomatology, which needs to be further explored with frequencies (e.g., role of infrasound) of wave-form and inner ear pressure measurements with surrogates. Currently, no standards exist to define limits on personnel acoustic exposures within these training environments, but the findings here indicate that prolonged exposure does seem to have an association with negative symptoms even in the absence of a medical diagnosis. It is possible that changes to training, modifications of the environment where the training is conducted (e.g., reconfigurations of the blast pits, etc.) and other subtle variations may be sufficient to mitigate a significant proportion of the risks that may be associated with acoustics.

Future work would be well-advised to focus attention on the longitudinal effects of significant acoustic exposures to track any potential changes in the experienced symptoms, frequency, or the intensity of the effects. Application of current methodology discussed here to longer duration investigations like those by Kubil et al. (17), may prove fruitful. The limitation of this work focusing on a single day data collection is known; however—it does not dismiss the need for in depth, longitudinal investigations of acoustic centric exposures in blast environments. Additionally, these longer-term investigations are needed to determine the permanence or transience of these disruptions and to understand if a cumulative effect exists such that repeated significant exposures reduces resiliencies and promotes heightened sensitivity to subsequent exposures, making individuals more vulnerable over time to exposures to additional OP and acoustic signatures. The limitation of the current study is a small sample size, this data needs to be further validated comprehensive assessment with objective metrics such as audiological assessment in a larger sample of subjects. Finally, efforts need to be made to expand the types of short-term effects investigated. Expanding current efforts to look more deeply at cognitive function and its association with bio-markers is critical to improving our understanding of OP.

## Data Availability

The datasets for this study will not be made publicly available because the datasets are of select military members and dates/locations might compromise subject identity.

## Author's Note

Material has been reviewed by the Walter Reed Army Institute of Research. There is no objection to its presentation and/or publication. The opinions or assertions contained herein are the private views of the author, and are not to be construed as official, or as reflecting true views of the Department of Defense. The investigators have adhered to the policies for protection of human subjects as prescribed in AR 70–25.

## Author Contributions

VS designed the study, analyzed the data, and assisted in writing. GK oversaw and assisted with field data collection of the study. CL supported analyses. JS, AM, MG, and AR collected the field data for this study. ME and VS wrote the manuscript and conducted analyses. JL oversees the lab and provided final editing and approval of the manuscript.

### Conflict of Interest Statement

The authors declare that the research was conducted in the absence of any commercial or financial relationships that could be construed as a potential conflict of interest.
